# N-Terminal Domain of Turkey Pancreatic Lipase is Active on Long Chain Triacylglycerols and Stabilized by Colipase

**DOI:** 10.1371/journal.pone.0071605

**Published:** 2013-08-16

**Authors:** Madiha Bou Ali, Aida Karray, Youssef Gargouri, Yassine Ben Ali

**Affiliations:** Laboratory of Biochemistry, National Engineering School of Sfax (ENIS), University of Sfax, Sfax, Tunisia; University of Graz, Austria

## Abstract

The gene encoding the TPL N-terminal domain (N-TPL), fused with a His6-tag, was cloned and expressed in *Pichia pastoris*, under the control of the glyceraldehyde-3-phosphate dehydrogenase (GAP) constitutive promoter. The recombinant protein was successfully expressed and secreted with an expression level of 5 mg/l of culture medium after 2 days of culture. The N-TPL was purified through a one-step Ni-NTA affinity column with a purification factor of approximately 23-fold. The purified N-TPL, with a molecular mass of 35 kDa, had a specific activity of 70 U/mg on tributyrin. Surprisingly, this domain was able to hydrolyse long chain TG with a specific activity of 11 U/mg using olive oil as substrate. This result was confirmed by TLC analysis showing that the N-TPL was able to hydrolyse insoluble substrates as olive oil. N-TPL was unstable at temperatures over 37°C and lost 70% of its activity at acid pH, after 5 min of incubation. The N-TPL exhibited non linear kinetics, indicating its rapid denaturation at the tributyrin–water interface. Colipase increased the N-TPL stability at the lipid-water interface, so the TPL N-terminal domain probably formed functional interactions with colipase despite the absence of the C-terminal domain.

## Introduction

Lipases are defined as triacylglycerol acylhydrolases (E.C.3.1.1.3) that catalyze the fats and oils hydrolysis at the oil-water interface to glycerol and free fatty acids.

Our understanding of the lipase action mode has made much progress as a result of the 3D structure resolutions of more than twenty lipases over the past few years [1].

The three-dimensional (3-D) structure of the human pancreatic lipase (HPL) consists of two functional domains [2]. The N-terminal domain belongs to the α/β hydrolase fold family of proteins [3] and contains the active site which involves a catalytic triad analogous to that present in serine proteases. A surface loop (Cys237–Cys261), the so-called lid or flap, prevents the substrate access to the active site in its closed conformation. HPL requires a small protein cofactor, colipase, for the enzyme to be able to bind water/triglyceride interface in presence of bile salt. Colipase binds to the C-terminal domain of HPL and exposes the hydrophobic tips of its fingers at the opposite side of its lipase-binding site [4].

In 2000, Sayari et al [5] purified turkey pancreatic lipase (TPL) from delipidated pancreases. This avian pancreatic lipase contains 450 amino acids and presents an experimental mass of 49665.31 Da [6]. Biochemical properties and kinetic studies were determined using emulsified system and monomolecular film techniques [5], [6].

TPL was expressed in *Pichia pastoris* [7]. In contrast to some previous studies showing that the heterologous expression could lead to a modification of the same biochemical properties [8], the recombinant TPL shows the same properties of the native TPL.

Contrary to the TPL [9] and horse pancreatic lipase (HoPL) [10], partial proteolysis assays to produce an active N-terminal domain failed using human pancreatic lipase HPL [11], pig pancreatic lipase (PPL) [12] and Ostrich pancreatic lipase (OPL) [9]. TPL cleavage by chymotrypsin generated three major fragments of about 35, 14 and 10 kDa, respectively. The N-terminal sequence of the 35 kDa fragment was the same as the native TPL. Based on its molecular mass (35 kDa), the C-terminal truncated TPL form would correspond to the N-terminal domain which was in favour of the degradation of the C-terminal domain upon chymotryptic cleavage [9].

The N-terminal TPL domain alone seems therefore to be active. This is in line with the fact that TPL whose N-terminal domain has a higher hydrophobic surface displays the highest interaction capacity with a lipidic substrate among the other pancreatic lipases [6].

The HPL N-terminal domain (N-HPL) produced in insect cells has a low tripropionine monomers (TC3) activity (48 U/mg), which could be explained by the absence of an open and stable flap, besides being inactive toward trioctanoine (TC8) and TC4 [11]. To study the role of the C-terminal domain in the HPL function, substitution and deletion experiments were performed [13].

Deletion of the C-terminal domain decreased both the amount of truncated mutant protein in the medium of transfected cells and the specific activity of the mutants. Still, maximum activity required colipase, indicating that the deletion mutants interacted with colipase. Jennens et al. [13] suggested that the C-terminal domain is required for the proper folding or processing of HPL to confer stability and increase activity, but is not absolutely required for the colipase reactivation of the bile salt-inhibited enzyme.

Deletion mutants of the C-terminal domain suggested that this region of HPL was not required for a functional interaction with colipase, but the C-terminal domain was critical for HPL maximal activity and stability [13].

To investigate the C-terminal domain deletion effect, and to study the biochemical properties of the N-terminal domain TPL (N-TPL), a high expression level system of the N-TPL is required for the study of the structure–function relationships of this protein. The methylotrophic yeast *Pichia pastoris* is a host system which has been widely used in both academic and industrial laboratories for the production of a variety of heterologous proteins [14].

In spite of a strong glycosylation of recombinant protein, the methylotrophic yeast *P. pastoris* has been successfully used for the recombinant expression of many foreign proteins [15]. This system involves many advantages, including the ability to integrate expression plasmids at specific sites and to grow cells at a high density [16]. Similarity to mammalian and insect cells, *P. pastoris* can carry out some co- and posttranslational modifications of foreign proteins and its products are usually obtained with the right disulfide bonds.

In this work, we report the expression of the N-terminal domain of TPL in *Pichia pastoris* to study the effect of the C-terminal domain deletion on the enzyme activity, and to verify if the N-terminal domain alone could interact and hydrolyze an insoluble substrate.

## Materials and Methods

### 1. Strains, Cell Culture and Vectors

The Escherichia coli strain XL1-Blue was used as a host for cloning the N-TPL PCR fragment in the *P.pastoris* transfer vector pGAPZαA (Invitrogen). The *P.pastoris* host strain was X33 (wild-type strain from Invitrogen). The *P.pastoris* transfer vector pGAPZαA (Invitrogen) was used for yeast transformation. The Pfu DNA polymerase, T4 DNA ligase, PCR purification kit and Midi-Prep Kit were purchased from Promega.

*Pichia pastoris* liquid cell cultures were grown in YPD medium containing 10 g yeast extract, 20 g Bacto-peptone and 20 g D-glucose. The YPDS medium was YPD medium to which 18.2 g sorbitol per liter were added. To prepare plates for solid cell cultures, 2% agar (w/v) were added to the YPD medium.

The Deoxycholic acid sodium salt (NaDC) (purity 99%) was purchased from Bio Basic Inc and Diethyl-p-nitro-phenylphosphate (E600) from Sigma-Aldrich-Fluka Chimie (St-Quentin-Fallavier, France).

### 2. Construction of the DNA Encoding the N-TPL Domain

Starting with TPL full-length DNA cloned into the pGAPZαA [7], which served as the template, the N-TPL mutant was obtained by PCR amplification using the following forward and reverse primers, both including a EcoRI restriction site (underlined):

Primer 1∶5′- GATCGAATTC TCTGAAGTTTGCTATGAC -3′

Primer 2∶5′- GATCGAATTC CCCCAAAGAGGAAAATCT- 3′

Primer 1 anneals with the TPL N-terminal sequence encoding the peptide (S, E, V, C, Y, D). Primer 2 anneals with an internal part of TPL DNA encoding the last five amino acid residues of the TPL N-terminal domain (R, D, F, P, L, W) The PCR reaction was carried out using pfu polymerase for 30 cycles with durations of 1 min at 95°C, 1 min at 60°C and 1 min 30 sec at 72°C. The PCR product was digested by the EcoRI restriction enzyme and inserted into the pGAPZαA vector previously digested by the EcoRI downstream of the GAP constitutive promoter as described by Sias [17]. Protoplasts of *E.coli* XL1-Blue were transformed with the ligation mixture using the chemical method [18] and the transformed clones were selected on Luria-Bertani (LB) plates containing 25 µg/ml Zeocin.

The recombinant *P.pastoris* expression vector (pGAPZαA/N-TPL) was propagated in the *E.coli* strain XL1-Blue and isolated using the Midi-prep purification system. The correct integration of the insert was checked by DNA sequencing.

### 3. Transformation of *P.pastoris* and Screening of N-TPL Secreting Transformants

Electrocompetent *P.pastoris* X-33 cells were prepared using standard methods [19] and their transformation was performed by electroporation according to Invitrogen manual. Prior to the yeast transformation procedure, recombinant vector (pGAPZαA/N-TPL) was linearized by the restriction enzyme BspHI. The recombinant yeast clones were selected on YPDS plates containing 100 µg/ml Zeocin. The colonies were subsequently screened by performing PCR reaction using as template the genomic DNA of the selected clones to confirm the integration of the pGAPZαA/N-TPL vector into the yeast genomic DNA [17].

Selected transformants were grown in 50 mL of YPD medium with 100 µg/ml Zeocin at 30°C under shaking at 150 rpm. Time course of N-TPL secretion in the culture media was determined for various clones.

### 4. Real-time PCR

The ICycler (Biorad) was used for Q-PCR amplification and detection. Q-PCR was prepared in 25 µl reaction mixture. Each reaction well contains 5 µl of template DNA (100 ng), 12.5 µl of SensiMix dT, 0.5 µl of SYBR Green I solution, 4 mM MgCl_2_ and 10 mM of forward (5′- CGTCCGTGTTGTAGGCGCTG -3′) and reverse (5′- CCTATTGCTGGGGCCATTCC -3′) primers to generate an amplicon of 276 pb. Serial 10-fold dilutions of plasmid DNA (pGAPZαA/N-TPL) were conduced to establish the standard curve. The negative control (without DNA template) was included in experimental runs. The Q-PCR cycling program was 2 min at 98°C for the activation of the hot-start enzyme, followed by 44 cycles of denaturation at 98°C for 5 s, annealing at 57°C for 10 s and elongation at 72°C for 10 s. Melting curves analysis was performed after completed Q-PCR collecting fluorescence between 65 and 95°C at 0.5°C increments.

### 5. Expression of His6-tagged N-TPL in *P. pastoris*

The most efficient N-TPL secreting selected transformant was pre-grown at 30°C in 250 ml shake flasks containing 50 ml YPD medium with 100 µg/ml Zeocin for 24 h to an OD600 of 4–6. This cell culture was further used to inoculate 500 ml shake flask containing 100 ml YPD medium without Zeocin. The production of N-TPL was conducted at 30°C for 48 hours with shaking at 150 rpm.

### 6. Purification of the His6-tagged N-TPL Protein

The culture was harvested by centrifugation (9500 rpm, 10 min, 4°C). The supernatant was then loaded onto an Ni–NTA affinity column (BioRad) previously equilibrated with buffer A (25 mM Tris pH 8; 2 mM Benzamidine). After washing the column with buffer A, the N-TPL fusion protein was eluted with 1 M imidazole.

### 7. Lipase Activity and Protein Assays

The lipase activity was measured titrimetically at pH 8.5 and 37°C with a pH-Stat using tributyrin (0.25 ml) in 15 ml of 2 mM Tris-HCl, pH 8, 150 mM NaCl or olive oil emulsion in the presence of 0.5 mM NaTDC and 0.1 mM CaCl_2._

Lipase activity was also measured using TC3 as substrate according to Ferrato et al. [20] or trioctanoyl (TC8) in the presence of 0.5 mM NaTDC and 0,1 mM CaCl_2_. One lipase unit corresponds to 1 µmol of fatty acid liberated per min.

Protein concentration was determined as described by Bradford [21] using BSA (E1% 1 cm = 6.7) as reference.

### 8. SDS-PAGE

Analytical polyacrylamide gel electrophoresis of proteins in the presence of sodium dodecyl sulfate (SDS-PAGE) was performed by the method of Laemmli [22]. The proteins were stained with Coomassie brilliant blue.

### 9. Effect of Temperature and pH on Enzyme Stability and Activity

Optimal pH and temperature were determined by examining the lipase activity at different temperatures and pH by pH-Stat assay using olive oil as substrate. pH stability was determined by incubating the lipase solution for 30 min at different pH at 4°C. Thermostability was determined by pre-incubating the enzyme at temperatures ranging from 37°C to 60°C. The lipase activity was measured after centrifugation under standard conditions.

### 10. Inhibition of the His6-tagged N-TPL by E600

N-TPL, either in the absence or in the presence of detergent (2 mM NaDC) was incubated with 4 mM E600 (diethyl para-nitro phenyl phosphate). The mixture was incubated at 4°C and the remaining lipase activity was measured using TC4 as substrate.

### 11. Interaction of the His6-tagged N-TPL with Colipase

To study the N-TPL interaction with Turkey colipase, N-TPL lipase activity was measured using TC4 as substrate in the presence and absence of colipase, in optimal conditions of pH and temperature.

### 12. Qualitative Analysis of Hydrolysis Products

Forty (40) micrograms of recombinant N-TPL were incubated with 25 mΜ of triolein emulsified in 1 ml buffer (2 mM Tris HCl, 100 mM NaCl 0.1 mM NaTDC and 0.5 mM CaCl_2_) at 37°C for 15 minutes. Lipolysis was stopped by adding 200 µl of 1 M HCl and mixing vigorously with 2 volumes of chloroform/methanol mixture (2∶1, v/v) in a 15 ml glass tube with a Teflon-lined screw cap. Lipids were immediately extracted as follows: after phase separation, the lower organic phase was transferred to a 15 ml test tube and dried over anhydrous magnesium sulphate. Once MgSO_4_ had precipitated, the clear organic phase was analysed by TLC. To separate lipid classes, 1 to 50 µl of lipid extracts were first spotted onto a thin-layer silica plate. The elution of the lipids was then performed in one step with a hexane:diethyl ether:methanol:acetic acid (82∶17∶0.3∶0.2) (v/v/v/v) solvent mixture.

Following chromatography, the plates were dried at room temperature for 10 min and lipid spots were visualized with iodine vapor.

### 13. Statistical Analysis

Experimental results were given as mean value±SD of three parallel measurements. Comparisons between values were analyzed by Student’s t test for unpaired data, and p<0.05 was considered significant. Statistical analysis was conducted using Microsoft Excel software.

## Results and Discussion

### 1. Construction of N-TPL Expression Vector

The DNA fragment encoding for the N-TPL (1014 bp) was amplified by PCR using plasmid DNA (pGAPZαA/TPL) as template with specific primers containing two EcoRI sites upstream of its first codon and downstream of its last codon. The amplified fragment was digested with EcoRI and inserted into the pGAPZαA vector previously digested with EcoRI, in frame with the yeast α-factor signal sequence at the N-terminal side for protein secretion and with the His6-tag sequence at the C-terminal side to facilitate the purification of the expressed protein. The integrity of the construction (pGAPZαA/N-TPL) was confirmed by DNA sequencing (data not shown).

### 2. Generation of Recombinant Clones Expressing N-TPL

The *P.pastoris* X33 strain was transformed by electroporation using (pGAPZαA/N-TPL) plasmid DNA linearized by the BspHI restriction enzyme. *P.pastoris* tranformants were selected on YPDS-Zeocin plates and incubated at 30°C for 3 days.

Two Zeocin-resistant clones picked on the solid selective medium were selected and the integration of N-TPL gene was analysed by PCR using the pGAPZαA universal primers (pGAP Forward and AOX1 primers). N4 and N6 clones showed the amplification of the expected size (1554 bp). This fragment corresponded to the size of the N-TPL gene (1014 bp) plus a portion of the vector (540 bp). This result confirms the integration of the expression cassette (pGAPZαA/N-TPL) in *P. pastoris* genomic DNA.

### 3. Selection of N-TPL-secreting Transformants

**Figure 1:**
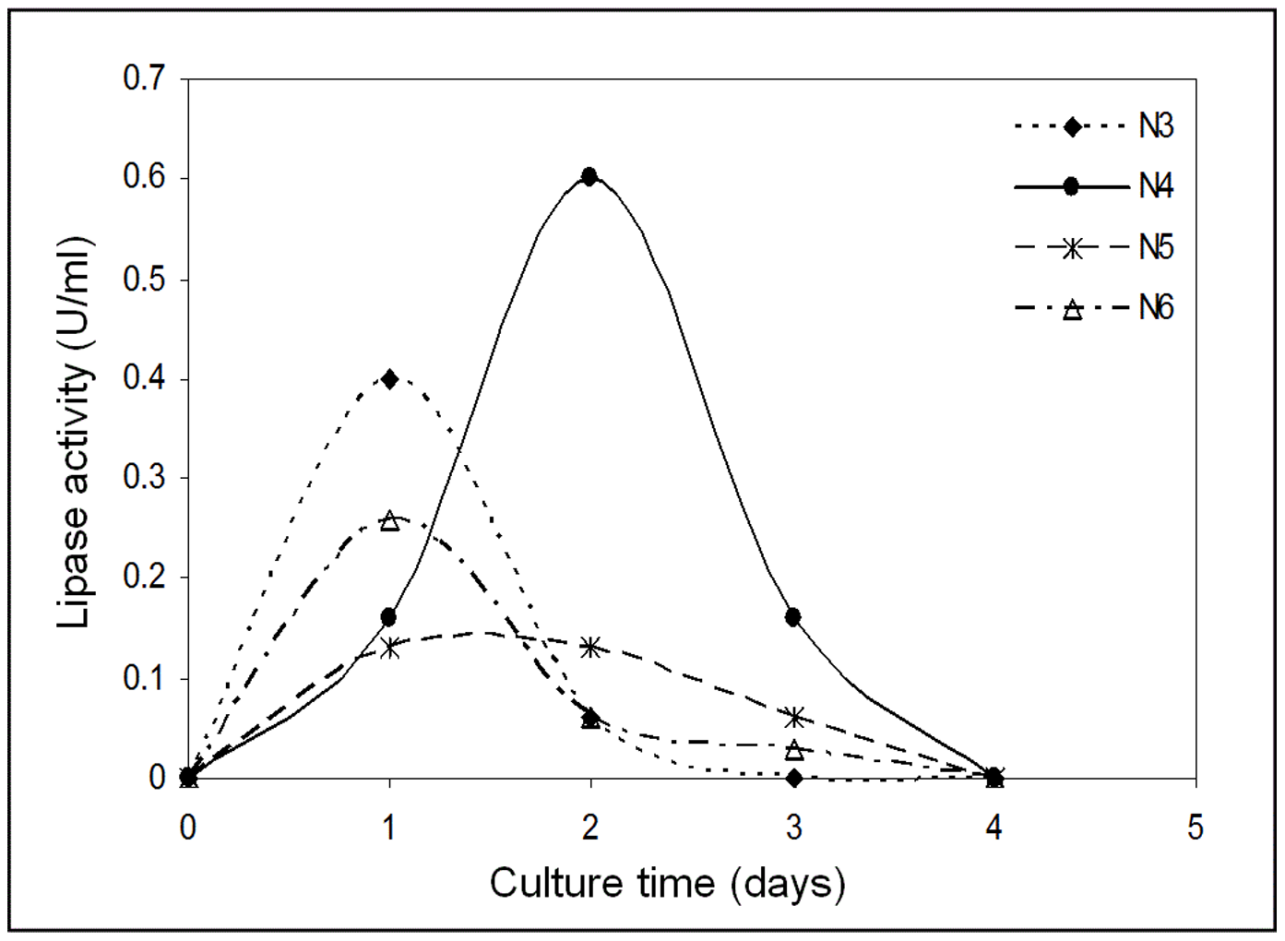
Time-course of the expression of His6-tagged N-TPL by four isolated clones of recombinant *Pichia pastoris*. The culture was carried out in 250 ml Erlen-meyer’s falsks with shaking at 150 rpm and 30°C.

Transformants carrying the N-TPL gene (N3, N4, N5 and N6) were grown on a YPD medium in 250 ml Erlen-meyer’s flasks with shaking at 150 rpm, 30°C to select the clone showing the highest lipase activity level. The time-course of the N-TPL secretion by the *P.pastoris* clones is shown in figure 1. As shown in this figure, the N4 clone exhibited the highest activity level reaching about 0.6 U/ml after 48 hours of culture. This clone was then selected for the production and purification of the recombinant N-TPL. Actually, there is no solid evidence that each integration event on the yeast genome contributes equally to the levels of the expressed protein. The multiple integration events have little detrimental effect on the expression of secreted protein in *Pichia pastoris* since other factors can also affect the expression level [23].

### 4. Analysis of N-TPL cDNA Copies Number in Selected Recombinant Strains by Q-PCR

**Figure 2:**
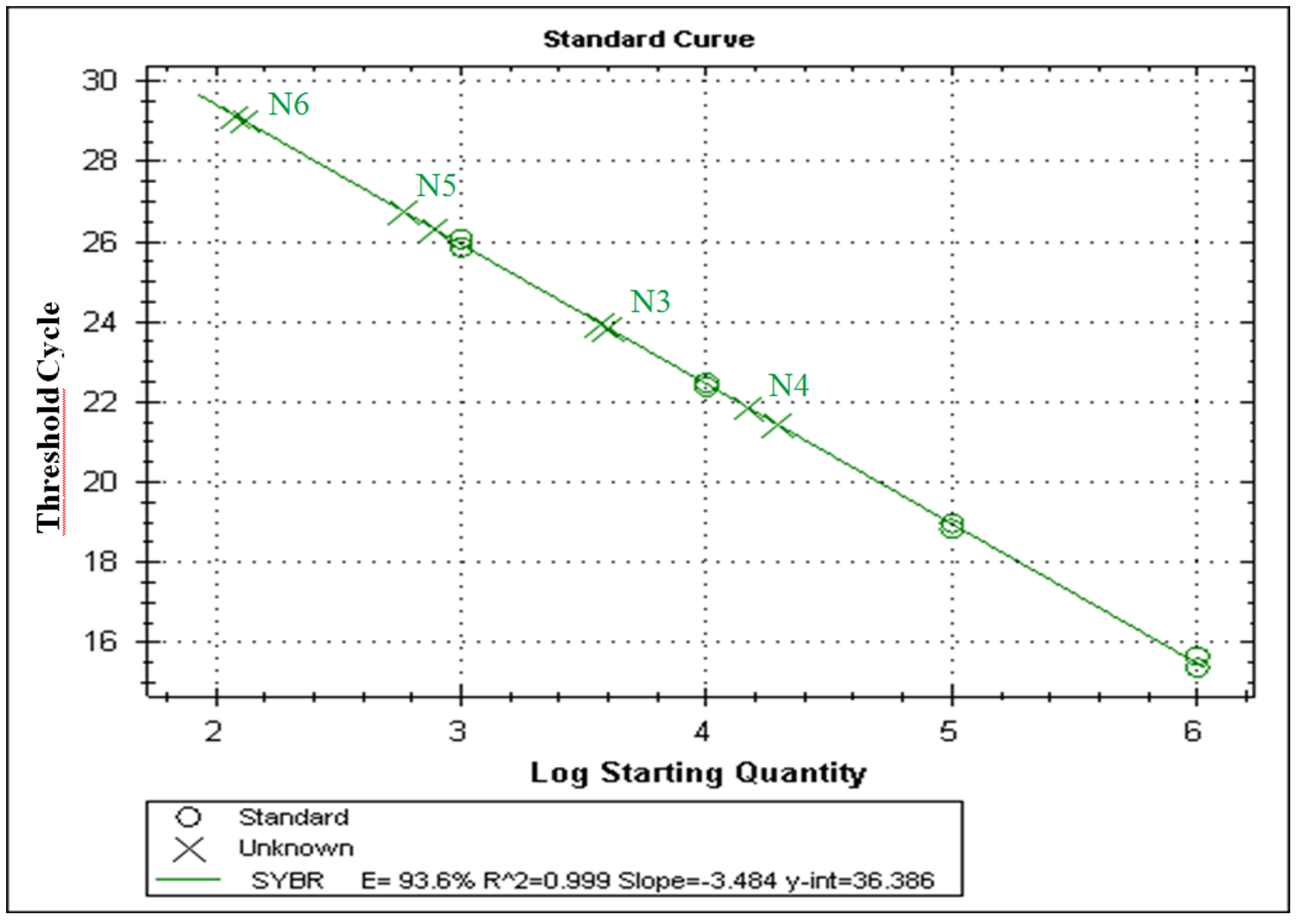
Q-PCR analysis of the N-TPL cDNA copies number in the genomic DNA of N3, N4, N5 and N6 clones. Correlation coefficient: 0.999; Slope: −3.484; PCR Efficiency: 93.6%.

Genomic DNA extracted from the four selected clones, was analyzed by Q-PCR to estimate the N-TPL cDNA copies number. As shown in figure 2, the N3, N4, N5 and N6 clones had a Ct value of 20, 18, 22 and 25 respectively. Based on the standard curve done with 10 fold serial dilution of plasmid DNA (pGAPZαA/N-TPL), the number of integrated expression cassette was estimated to 4 copies for N3 and N4, 3 copies for N5 and 2 copies for N6. These results are in agreement with the difference in the activity levels of these clones (figure 1). In fact, N5 and N6 exhibited the lowest lipase activity level compared to the other clones.

### 5. Purification of the N-TPL

After 48 h of yeast culture, the corresponding amount of recombinant N-TPL was estimated to be around 5 mg/l of culture medium.

The N-TPL was purified using a one step Ni-NTA affinity column. It was eluted from the column at an imidazole concentration of 1M in buffer A.

In the culture supernatant, the N-TPL specific activity was found to be 3 U/mg when using TC4 as substrate. The specific activity of the purified N-TPL reached 70 U/mg under optimal conditions of temperature and pH (37°C and pH 8.5) and in the presence of 0.5 mM NaTDC; 0.1 mM CaCl_2_. Starting with the whole *P.pastoris* culture supernatant, 23-fold purification was achieved and the overall recovery, based on enzyme activity, was 25% (Table 1).

**Table 1:** Flow sheet of His6-tagged N-TPL purification.

Purification step	Total activity (U)	Specific activity (U/mg)	Yield (%)	Fold
Supernatant	40	3	100	1
Ni-NTA affinity chromatography	10	70	25	23.33

**Figure 3:**
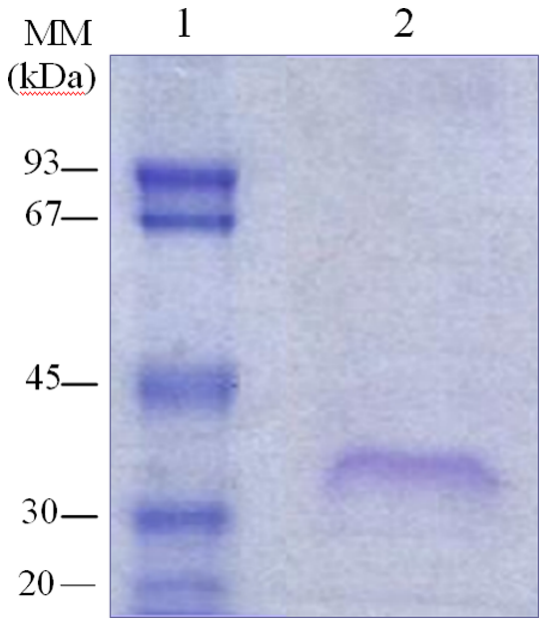
SDS-PAGE of the purified His6-tagged N-TPL performed on 13% acrylamide gel. Lane1: Low molecular weight marker; Lane 2∶30 µg of purified His6-tagged N-TPL.

The purification effectiveness was monitored by SDS-PAGE, showing that the purified N-TPL appeared as a single band with an apparent molecular weight of around 35 kDa (figure 3).

### 6. Effect of Temperature on the N-TPL Stability

**Figure 4:**
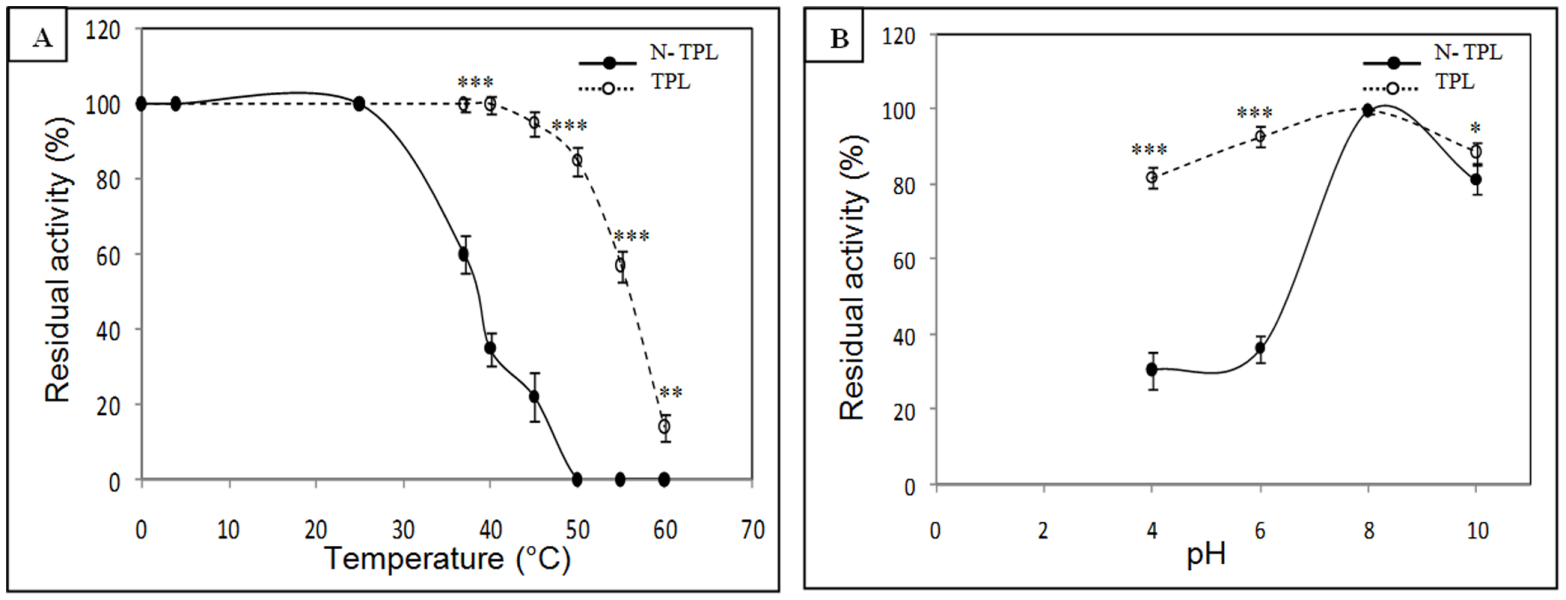
Stability of His6-tagged N-TPL and TPL. Effect of temperature (**A**) and pH (**B**). His6-tagged N-TPL was incubated at different temperatures and pH for 5 min and TPL was incubated at different temperatures and pH for 30 min. The activity is measured under optimal conditions (without colipase) and using TC4 as substrate. 100% activity corresponds to AS = 70 U/mg and AS = 9500 U/mg for His6-tagged N-TPL and TPL, respectively. Bars represent means ± SD. *p<0.05, **p<0.01, ***p<0.001 versus TPL.

In order to study the N-TPL thermal stability, the whole TPL and N-TPL were incubated at different temperatures for 5 min at pH 8 (figure 4A).

As shown in figure 4A, the N-TPL was stable for 5 min at temperatures below 25°C and it retained 60% of its activity after 5 min of incubation at 37°C. At temperatures over 37°C, the activity deceased dramatically and the N-TPL lost its activity after 5 min of incubation at 50°C. Therefore, it was unstable at temperatures over 37°C unlike the TPL which kept 100% of its activity after 30 min incubation at 37°C and retained 95% of its activity at 50°C [7]. These results show that the deletion of the C-terminal domain of the TPL decreases the thermal stability of the protein. The C-terminal domain is therefore required for the thermal stability of the enzyme.

### 7. Effect of pH on the N-TPL Stability

In figure 4B, stability at different pH was studied for both N-TPL and the whole enzyme (TPL). In acid pH, the N-terminal domain appeared to be less stable then the TPL. The latter kept around 80% of its initial activity after 30 min of incubation at pH 4, whereas the former lost around 70% of its initial activity after 5 min of incubation.

When incubating in basic pH, the TPL and the N-terminal domain kept 85% and 80% of their activity after incubation of 30 and 5 min, respectively, at pH 10.

This low pH stability could be explained by the absence of the C-terminal domain which may have effects on the conformational change of the N-terminal domain.

### 8. Activity of N-TPL on Different Substrates

To investigate the effect of the absence of C-terminal domain on the N-TPL catalytic activity, the specific activity of this protein was measured using different substrates: soluble ones (tripropionin (TC3), tributyrin (TC4)) and insoluble substrates (trioctanoin (TC8) and olive oil (OO)) under the optimal conditions of each assay. These values were compared with those of TPL (Table 2).

**Table 2:** Specific activity of His6-tagged N-TPL and TPL on different substrates.

	TC3	TC4	TC8	O.O
TPL	900±20	9500±150	1800±50	5300±100
His6-tagged N-TPL	65±4	70±4	40±2	11±1

The activity of N-TPL was taken from the linear portion of the titration curve.

The pure N-TPL had specific activities of 70, 65, 42 and 11 U/mg using TC4, TC3, TC8 and OO as substrates, respectively.

These results indicate that the N-TPL is more active on the soluble short-chain substrates (TC4 and TC3) than on insoluble substrates (TC8 and OO). Nevertheless and despite the deletion of the C-terminal domain, the N-terminal domain of TPL is still able to hydrolyses alone completely insoluble substrates, which is the main characteristic to define the lipase family.

This property wasn’t conserved in the case of the human pancreatic lipase N-terminal domain (N-HPL) produced by proteolytic digestion. This domain was active on TC3 (48 U/mg) but inactive toward TC4, TC8 and OO [11]. The low activity of the N-TPL could be explained by the absence of the C-terminal domain and therefore the absence of the ß_5_’ loop which interact with the interface and the flap which is not stabilised under its opened form. These structural modifications could reduce the hydrophobic surface involved in the stabilization of the enzyme on the lipid interface [24].

### 9. Kinetic Studies of the N-TPL Activity on Tributyrin

Despite the high energy existing at the tributyrin–water interface, which was described as a cause for the irreversible denaturation of several lipases, TPL is able to hydrolyze TC4 emulsion efficiently without interfacial denaturation [25].

**Figure 5:**
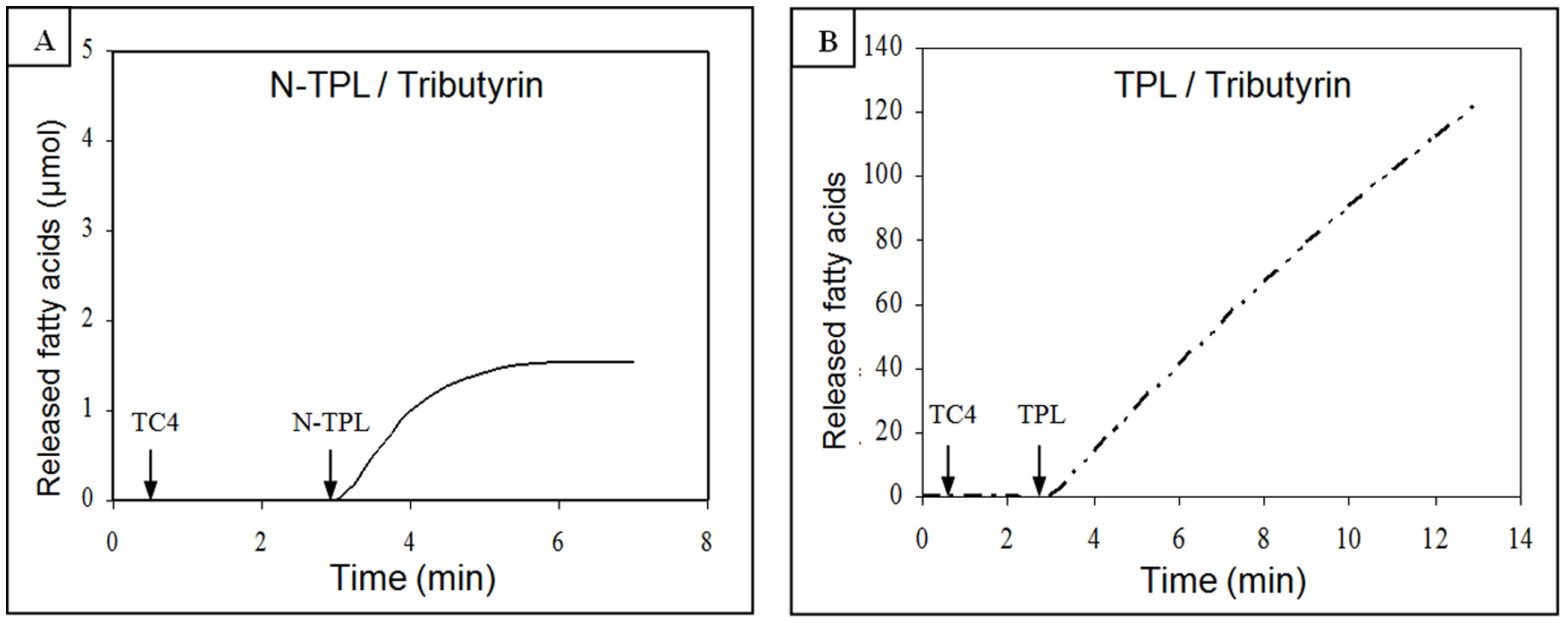
Hydrolysis kinetics of tributyrin by His6-tagged N-TPL and TPL. (**A**) His6-tagged N-TPL hydrolysis kinetic. (**B**) TPL hydrolysis kinetic. Lipolytic activity was followed at pH 8.5 and at 37°C in the presence of 0.5 mM NaTDC and 0.1 mM CaCl_2_ (without colipase).

To study the N-TPL behavior at the tributyrin–water interface, we followed the kinetics of TC4 hydrolysis under optimal conditions of temperature and pH. As shown in figure 5A, the N-TPL exhibited a kinetics that remained linear for only 1 min in the presence of 0.5 mM NaTDC and 0.1 mM CaCl_2_. However, the TPL presented linear kinetic remaining for 6 min in the absence of any tensio-active reagent (figure 5B).

These results indicate that the deletion of the C-terminal domain containing the β5′ Loop playing a critical role in the lipase lipid interactions [24] is accompanied by the rapid N-TPL denaturation on the tributyrin–water interface.

### 10. Interfacial Activation of N-TPL

**Figure 6:**
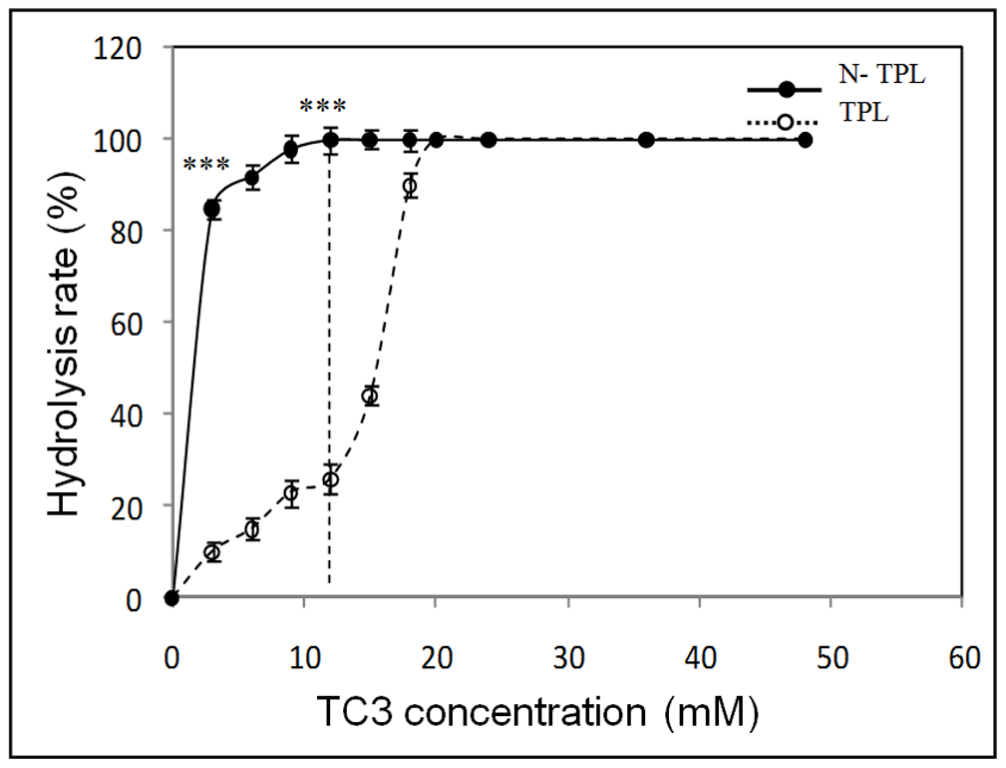
Hydrolysis rate of TC3 by His6-tagged N-TPL and TPL as a function of substrate concentration. The TC3 solutions were systematically prepared in 30 mL of 0.33% GA and 0.15 M NaCl. The CMC of TC3 (12 mM) is indicated by vertical dotted line. Bars represent means ± SD. *p<0.05, **p<0.01, ***p<0.001 versus TPL.

The interfacial activation is a phenomenon observed for some lipases under some particular experimental conditions [26]. though, the interpretation of this experiment is controversial [20], it can still give an idea on lipases affinity to soluble or insoluble aggregated substrate. The hydrolysis rates of TC3 emulsified in 0.33% GA and 0.15M NaCl by N-TPL and TPL as a function of substrate concentration are shown in figure 6. In contrast to TPL that slowly hydrolysed TC3 when it was presented in the water-soluble state and up to the solubility limit of TC3 (12 mM), the TPL activity increased rapidly to reach its full activity, indicating that it presented the interfacial activation phenomenon [5]. Contrary to the TPL, the N-TPL reached its maximum specific activity before the solubility limit of TC3, which indicates the loss of the interfacial activation phenomenon after the C-terminal domain deletion. The disappearance of the interfacial activation phenomenon could be explained by an intermediate conformation between the closed (inactive) and open (fully activated) conformations of N-TPL.

In fact, a not fully opened conformation, in which the active site remained inaccessible to the solvent, was observed for Thermomyces lanuginosa lipase. [27].

Furthermore, Ranaldi et al suggested the presence of an intermediate but still closed conformation of the HPL lid which might then evolve toward the open conformation in a more favorable way than the closed one. [28].

### 11. N-TPL Inhibition by E600

**Figure 7:**
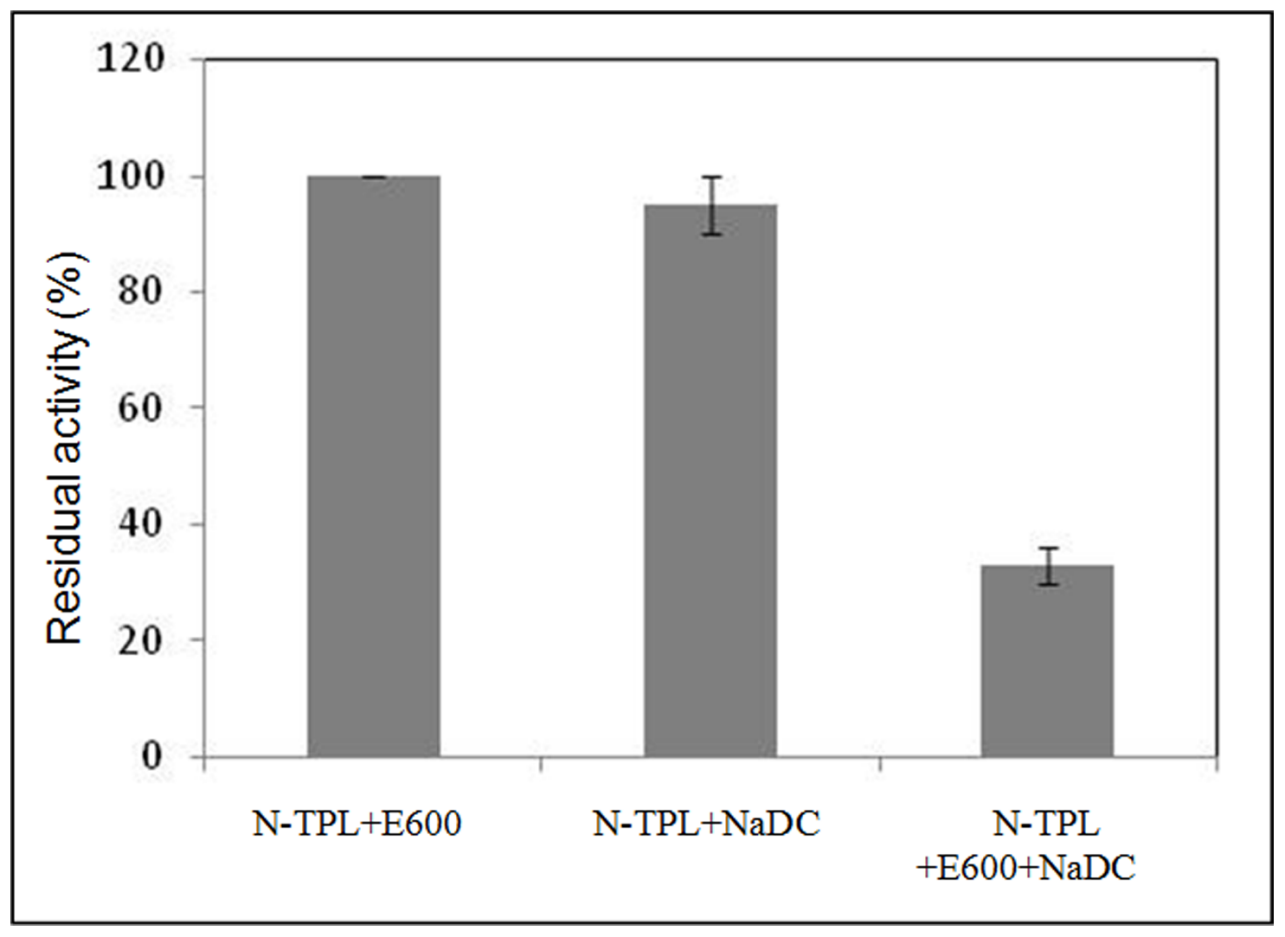
Effect of E600 on the activity of His6-tagged N-TPL. The protein was incubated with 4 mM E600, in the presence and absence of NADC (2 mM), using TC4 as substrate. Activity is measured at 37°C and pH 8.5.

To study the effect of E600, a serine inhibitor soluble in water, on the activity of N-TPL we incubated the protein with E600 in the presence and in the absence of (NaDC) (2 mM), then we measured the N-TPL activity on TC4 under optimal conditions. As shown in figure 7, N-TPL was not inhibited by E600 in the absence of NaDC. When NaDC was added to the incubation media in the presence of E600, N-TPL retained only 36% of its activity after 3 min of incubation at 4°C.

These results could be explained by the fact that in the presence of E600 alone, the N-TPL lid domain is in a position covering the catalytic site, this preventing the access of E600 to the catalytic serine. When bile salts are added, the E600 forms mixed micelles with NaDC which forms an interface where N-TPL will be adsorbed, its lid domain will be fully opened and the catalytic serine will be accessible to the inhibitor. This result shows that the N-TPL serine is not fully accessible, which might be explained by an intermediate position of the lid domain [27] [28].

### 12. Interaction of N-TPL with Colipase

**Figure 8:**
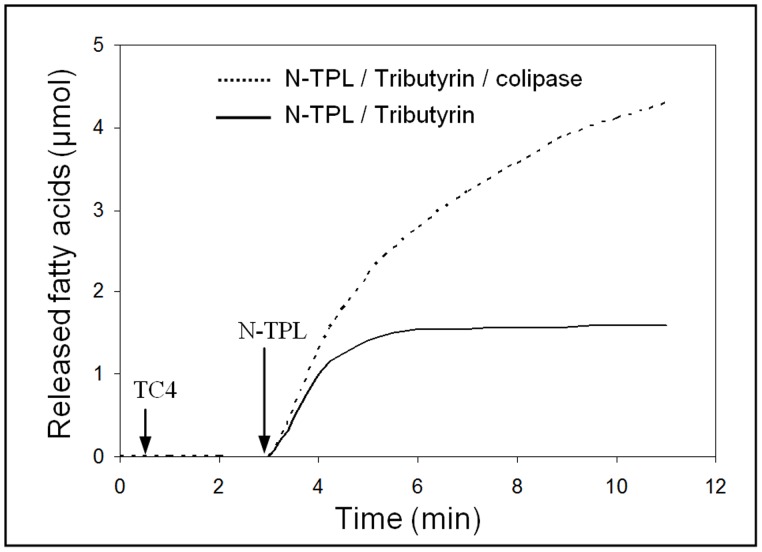
Hydrolysis kinetic of tributyrin by His6-tagged N-TPL in the presence of colipase. Lipolytic activity was followed at pH 8.5 and at 37°C.

In order to know if there is an interaction between the N-TPL and colipase, we measured the activity of this enzyme using TC4 as substrate under optimal conditions of temperature and pH in the presence and in the absence of colipase (figure 8).

We found that, in the absence of colipase, the N-TPL lost its activity after 3 min of hydrolysis, whereas in the presence of colipase, N-TPL remained active even after 8 min of hydrolysis. These results show that colipase increases the N-TPL stability at the lipid-water interface.

These findings can be explained by the fact that colipase may establish low energy interactions with opened lid in the N-terminal domain [29] despite the absence of the entire C-terminal domain. Similar results were obtained by Jennens et al who showed that the deletion of the C-terminal domain may affect substrate binding sites in the N-terminal domain and that this binding was partially restored by the addition of colipase [13].

It is worth noticing that, unlike bile salt inhibited TPL which is reactivated by injection of colipase in the reaction medium, the bile salt inhibited N-TPL is not reactivated by colipase (data not shown).

In fact, Procolipase did bind to the N-terminal domain of HPL in the crystals of the complex formed in the presence of mixed micelles, but contacts with the C-terminal domain were maintained and thought to be important components of the binding reaction [29]. The interaction of colipase with the N-terminal domain of lipase is analogous to the binding of the N-terminal domain of lipoprotein lipase with its protein cofactor, ApoCII [30], [31].

We also found an increase of 15% in the N-TPL activity when colipase was present in the hydrolysis medium. This effect is not so important when compared with that of the intact TPL with colipase. This result could be explained by the fact that the N-TPL/colipase interaction is not very stable, therefore the enzyme is not stabilized in its adsorbed form (E*) due to the absence of the C-terminal domain containing colipase binding sites.

### 13. Qualitative Analysis of Triolein Hydrolysis Products by TLC

To confirm the capacity of N-TPL to hydrolyze the insoluble substrates, TLC analysis of the triolein hydrolysis products was carried out.

**Figure 9:**
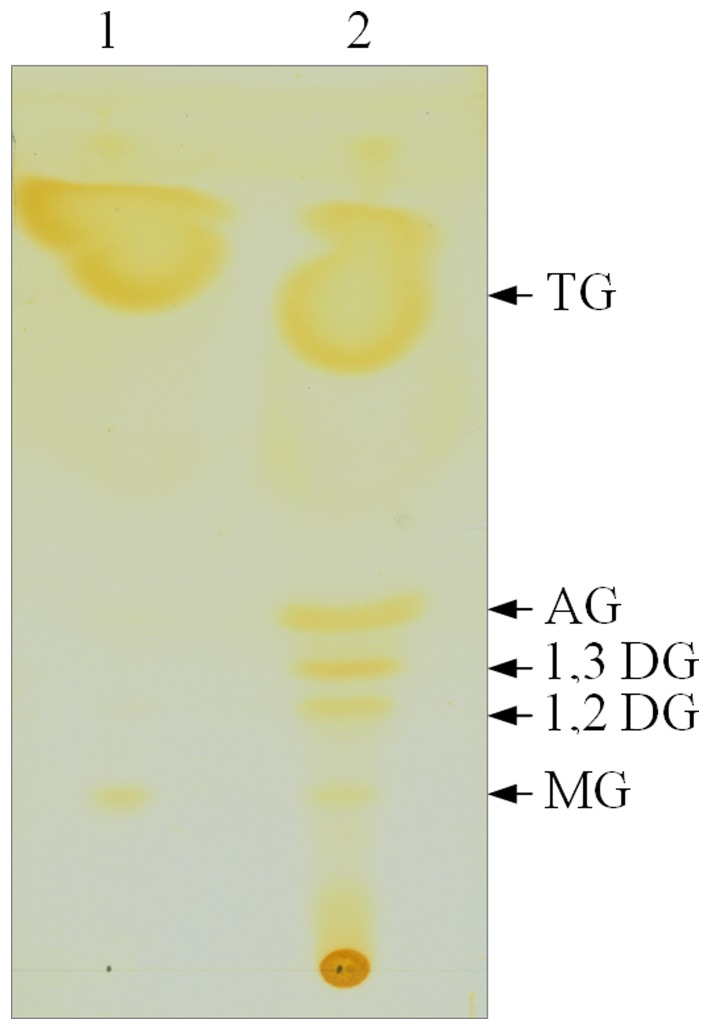
TLC analysis of hydrolysis products of triolein by His6-tagged N-TPL. Lane1: reaction products after 15 min incubation at 37°C of triolein in the absence of enzyme. Lane2: reaction products after 15 min incubation at 37°C of Triolein in the presence of His6-tagged N-TPL (14 µg). Reaction products were extracted with Chloroform/methanol mixture (2∶1, v/v).

As shown in figure 9, the N-TPL hydrolyses triolein efficiently and liberates products: diacylglycerol, monoacylglycerol and fatty acids. This result confirms that the N-TPL is able alone to catalyze the hydrolysis of the insoluble substrates.

### Conclusion

In this paper, we investigated the TPL N-terminal domain expression in *Pichia pastoris* to study the effects of the C-terminal deletion effects on the N-TPL activity, stability and some other biochemical properties.

The N-TPL was produced at a level of 5 mg/l of culture medium. The purified protein has a specific activity of 70 U/mg, 40 U/mg and 11 U/mg on tributyrin, trioctanoin and emulsified olive oil, respectively. Our results show that despite the absence of the C-terminal domain, the N-TPL continues to hydrolyse long chain triacylglycerol. Previous studies show that the N-HPL is inactive toward medium chain substrates [32]. Unlike the whole TPL, the N-TPL becomes unstable towards temperature and acidic pH after the deletion of the C-terminal domain leading to the conformational change of the N-terminal domain.

The recombinant N-terminal domain presents non linear kinetics which can be explained by the rapid denaturation of the N-TPL at the tributyrin-water interface. The deletion of the C-terminal domain contains the β5′ Loop playing a critical role in the lipase lipid interactions [24]. But, despite the absence of the C-terminal domain, the TPL N-terminal domain formed functional interactions with colipase, which increases the stability of the N-TPL at the lipid-water interface. These interactions are not so stable due to the absence of the C-terminal domain containing colipase binding sites.

Our results showed that the deletion of the C-terminal domain has a negative effect on the activity and stability of TPL, but this domain is not absolutely required to allow the N-TPL to hydrolyze the long chain substrate and to interact with colipase.
